# Aortic dissection is a disease of the vasa vasorum

**DOI:** 10.1016/j.xjon.2020.12.012

**Published:** 2021-01-06

**Authors:** Axel Haverich, Erin C. Boyle

**Affiliations:** Department of Cardiothoracic, Transplantation, and Vascular Surgery, Hannover Medical School, Hannover, Germany

**Keywords:** dissection, intramural hematoma, vasa vasorum

**Figure:**
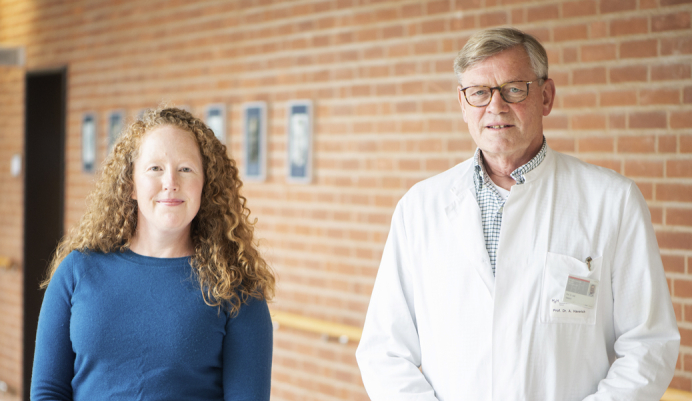
Erin C. Boyle, PhD (*left*), and Axel Haverich, MD (*right*)

**Central Message**

We propose that intramural hematoma and dissection always progress from the outside-in, with vasa vasorum dysfunction playing a crucial role in disease etiology.

See Commentary on page 33.

***Associate Editor's Introduction—**Understanding the initiating event of an acute aortic syndrome is made difficult by the inability to observe the process play out in the early stages of development; clinically, we are left with the result of whatever occurred. A “grand unified theory” of aortic disease would ideally link acute aortic dissection (AAD) and intramural hematoma (IMH) in a continuum of the same process (and, for that matter, penetrating aortic ulcers). It is easy to accept that IMH is a variant of AAD when we are unable to identify the primary intimal tear. In this review, the authors have flipped the paradigm and propose essentially that AAD is a variant of IMH in which the IMH has extended into and through the intima. The proposed mechanism centers on the vasa vasorum, an anatomic structure largely ignored but potentially a new focus of research and, eventually, clinical intervention.*

**Abe DeAnda Jr, MD**

Textbook knowledge and medical guidelines would suggest that aortic dissection presenting with an intimal tear and intramural hematoma (IMH) represent different disease entities. Current dogma dictates that IMH is pathologically distinct from classical dissection, with dissection being initiated in the intimal layer and IMH beginning in the tunica media. For spontaneous dissections of the aorta or in peripheral arteries, we believe that there is ample evidence for a common pathomechanism in the initiation of both dissection and IMH, with disease always affecting the medial layer first. Thus, similar to what we have proposed for atherosclerosis development,^1^^,^^2^ we believe that both IMH and dissection always progress from the outside-in, with vasa vasorum dysfunction playing a crucial role in disease etiology and pathogenesis.

Vasa vasorum are blood microvessels responsible for nutrition and oxygenation of the adventitia and outer media of medium- and large-sized vessels. Dysfunction of vasa vasorum by either malperfusion or leakiness has been shown to precipitate and exacerbate atheroma formation^1^^,^^2^ in a process termed the outside-in progression of atherosclerosis. Like atherosclerosis, IMH and dissections show site specificity. Both disease entities are restricted to large- and medium-sized arteries possessing vasa vasorum. In particular, vasa vasorum are the most abundant in the ascending aorta and arch—exactly the sites most susceptible to the development of aortic dissections. Although not all arteries possessing vasa vasorum are initiation sites for IMH or dissections, these 2 disease entities do share the same predilection sites.

It is generally accepted that rupture of vasa vasorum and hemorrhage directly into the tunica media is the inciting event in IMH. However, it remains a significant point of contention whether the causal event in dissection is an initial intimal tear that allows blood to accumulate in the media (inside-out mechanism) or whether it also involves disruption of vasa vasorum and a subsequent secondary tear of the intima overlying the hemorrhage (outside-in mechanism). These 2 primary theories on the pathogenesis of aortic and peripheral artery dissection were recently presented in separate position papers on spontaneous coronary artery dissection by the American Heart Association^3^ and the European Cardiology Association.^4^

Careful histological investigation has suggested that the tunica media is the most vulnerable arterial layer in both IMH and dissection, with vasa vasorum dysfunction playing a major role. Looking back over a century, the first (accessible) report of aortic dissection without an intimal tear was presented by Krukenberg in 1920.^5^ Krukenberg was the first to postulate that rupture of vasa vasorum was involved in IMH formation and “only in the end is there perforation into the lumen.” In 1931, the Yale pathologist Dawson Tyson published a case series of aortic dissections, several of which did not show evidence of an intimal tear.^6^ Similarly in 1953, Albert Hirst and colleagues published autopsy findings of 505 aortic dissection cases in which 4% had no identifiable intimal tear.^7^ In the early 1950s, Hirst and his contemporary Gore^8^ really championed the idea that aortic dissections began with hemorrhage from vasa vasorum and that an intimal tear, which was usually present, was only secondary to intramural dissection. Recently, this hypothesis has begun to gain favor again.^9^^,^^10^ Thorough histopathological analyses have demonstrated that most (95%) aortic dissection tears originate in the outer third of the media alongside the vasa vasorum.^9^ In most (but not all) cases, this leads to the development of a secondary tear of the intima overlying the hemorrhage. We would argue that these observations support the conclusion that IMH and dissection represent the same disease at different stages. In fact, in neuroscience, researchers and clinicians do not distinguish between IMH and dissection with intimal tear—they simply call both entities spontaneous cervical arterial dissection. The incidence of IMH without intimal tears clearly points to an outside-in progression of disease for arterial dissections. Therefore, dysfunction of the adventitial and medial microcirculation is a common denominator in disease initiation and progression, with intimal layer damage as only the final consequence.

There is increasing evidence that vasa vasorum dysfunction is a common pathophysiological link between IMH and dissection. Rupture of vasa vasorum and bleeding into the media is the most obvious path to the formation of IMH and subsequent dissection. On the other hand, malperfusion of the vessel wall microvasculature through the compressive forces of hypertension can lead to hypoxic conditions within the outer media. In large animal models in which aortic vasa vasorum flow has been disrupted, the outer third of the tunica media quickly becomes hypoxic and undergoes ischemic necrosis and degenerative alternations that are hypothesized to contribute to the development of aortic dissection.^11^^,^^12^ Hypoxic conditions in the media are likely to stimulate angiogenesis of vasa vasorum neovessels, which are by their very nature fragile and leaky. Therefore, hemorrhage from these neovessels also may contribute to IMH and dissection. Aortic dissections frequently progress very rapidly to involve the entire aorta. In contrast, IMH usually involves only a limited area of the aorta. This can be explained by the different flow and pressure conditions within the aortic wall once the intimal layer has torn. At that point, full aortic pressure is delivered to the tissue within the medial layer via the aortic lumen, whereas tissue pressure in IMH may remain low after rupture of vasa vasorum alone.

There are several shared risk factors that predispose individuals to IMH or aortic dissection, suggesting similar underlying pathomechanisms. Chronic hypertension and acute elevation of blood pressure precipitated by stress or substances (eg, cocaine, methamphetamines) are significant risk factors for IMH and dissection.^13^^,^^14^ Similar to the acute events of atherosclerosis (ie, stroke, myocardial infarction), there is also significant seasonal variation in aortic and cervical artery dissections.^15^^,^^16^ In these cases, seasonal variation in particulate air pollution and infectious diseases has been implicated. Also similar to atherosclerosis, inflammatory mechanisms have been increasingly recognized to play an important role in IMH and dissection.^17^, ^18^, ^19^ Elevated systemic inflammation is associated with aortic dissection, and significant infiltration of inflammatory cells (primarily macrophages) has been observed within the adventitial and medial layers of dissected areas in proximity to the vasa vasorum. In our histological observations of 70 surgical specimens of the ascending aorta obtained during elective repair of asymptomatic patients, we also observed vessel wall inflammation in more than one-half of the specimens (unpublished observation). Inflammatory cells were located primarily in the adventitia and the medial layer of the aortic wall, with the intimal layer generally spared. The common risk factors shared by IMH, dissection, and atherosclerosis strongly suggest a common pathomechanism in disease initiation and/or progression. We believe that the risk factors affect the arterial wall microvasculture, and thus dysfunction of the vasa vasorum is the unifying factor in all 3 diseases.

In summary, we propose that IMH and dissection including an intimal tear represent the same disease at different stages for the following reasons: (1) they occur at the same distinct anatomic sites; (2) malfunction of vasa vasorum has been shown for both; (3) clinically, IMH often propagates to dissection, suggesting 2 stages of a single disease; (4) like aortic aneurysms, both are characterized by vascular wall inflammation and media necrosis; and (5) IMH and aortic dissection with intimal tear share common risk factors.

Currently, IMH and dissection are considered distinct disorders, and thus there are differential guidelines with respect to therapeutic approaches.^3^^,^^4^ However, we argue that IMH likely represents an early stage of aortic dissection that may or may not propagate to classical aortic dissection by producing an intimal tear, which would always occur in a reverse fashion. Future research is needed to confirm this concept through systematic clinical observation, histopathological analysis, and, potentially, animal experiments. The striking similarity to the pathogenesis of atherosclerosis combined with the similar histological findings regarding cellular infiltrates in aortic aneurysms should initiate further research on the pathogenesis of aortic pathologies in general. In addition, these findings must be integrated into the growing understanding of the pathogenesis in arterial dissections affecting other vascular territories, such as cervical and coronary artery dissection. Only then will we be able to propose preventive measures and specific medications to individuals at risk. If this concept can be supported by further clinical and animal model studies, then diagnostic, preventive, and therapeutic measures can focus on the testing, prevention, and restoration of microvascular function in future clinical practice.

### Conflict of Interest Statement

The authors reported no conflicts of interest.

The *Journal* policy requires editors and reviewers to disclose conflicts of interest and to decline handling or reviewing manuscripts for which they may have a conflict of interest. The editors and reviewers of this article have no conflicts of interest.
